# Mental health and psychosocial interventions in the context of climate change: a scoping review

**DOI:** 10.1038/s44184-024-00054-1

**Published:** 2024-03-12

**Authors:** Siqi Xue, Alessandro Massazza, Samia C. Akhter-Khan, Britt Wray, M. Ishrat Husain, Emma L. Lawrance

**Affiliations:** 1 Department of Psychiatry, Temerty Faculty of Medicine, University of Toronto, Toronto, ON Canada; 2 Centre for Global Mental Health, The London School of Hygiene & Tropical Medicine, London, UK; 3 Department of Health Service & Population Research, Institute of Psychiatry, Psychology, & Neuroscience, King’s College London, London, UK; 4 Department of Psychiatry and Behavioral Sciences of Stanford Medicine, Stanford, US; 5 Campbell Family Mental Health Research Institute, Centre for Addiction and Mental Health, Toronto, ON Canada; 6 Institute of Global Health Innovation, Imperial College London, London, UK; 7 Grantham Institute - Climate Change and the Environment, Imperial College London, London, UK; 8 Mental Health Innovations, London, UK

**Keywords:** Planetary science, Psychology, Health care

## Abstract

The evidence on the impacts of climate change on mental health and wellbeing is growing rapidly. The objective of this scoping review is to understand the extent and type of existing mental health and psychosocial interventions aimed at addressing the mental health and psychosocial impacts of climate change. A scoping review methodology was followed. MEDLINE, PsycINFO, and Web of Science databases were searched from inception to May 2022. Comprehensive gray literature search, including expert consultation, was conducted to identify interventions for which peer-reviewed academic literature may not yet be available. Data on intervention type, setting, climate stressor, mental health outcome, evaluation, and any other available details were extracted, and results were summarized narratively. Academic literature search identified 16 records and gray literature search identified a further 24 records. Altogether, 37 unique interventions or packages of interventions were identified. The interventions act at the levels of microsystem, mesosystem, exosystem, and macrosystem through diverse mechanisms. While most interventions have not been formally evaluated, promising preliminary results support interventions in low- and middle-income-country settings disproportionately affected by climate disasters. Interventions from multidisciplinary fields are emerging to reduce psychological distress and enhance mental health and wellbeing in the context of climate change. This scoping review details existing evidence on the interventions and summarizes intervention gaps and lessons learned to inform continued intervention development and scale-up interventions.

## Introduction

Climate change is increasingly recognized as a public health emergency^1,2^. Beyond its well-recognized physical health consequences, the crisis impacts mental health in profound ways^1,3–5^. Climate events have been associated with worsened psychiatric mortality outcomes, including depression, post-traumatic stress disorder (PTSD), and suicide^6^. Various population groups have been identified as being particularly at-risk, including children and youth, older adults, pregnant women, people living with chronic illnesses, and racialized communities^6,7^. Indigenous groups worldwide have described feelings of sadness, anger, grief, fear, and helplessness from climate change-related forced migration, disrupted cultural continuity, and historical and ongoing disempowerment^8^.

Researchers in the climate change and mental health space acknowledge the intricate tension between recognizing the detrimental mental health impacts of climate change while not pathologizing culture-specific, expected, and adaptive responses to ongoing and anticipated threats. To encapsulate the nuanced range of experiences, new vocabulary, such as climate emotions, eco-anxiety, and ecological grief, have been introduced to literature^4,7^. Academics and third-sector organizations have also begun to identify protective coping mechanisms, including “active hope” and “meaning-focused coping” that emphasize acting in line with values, developing positive framings, and creating hope through action and trust in societal actors^9–11^.

Despite the accumulating evidence on climate-related mental health and/or psychological responses and coping strategies, little is known about evidence-based interventions to mitigate the negative consequences and support individuals and their communities. Of the 120 original studies identified in a scoping review on climate change and mental health research, the vast majority were cross-sectional studies quantifying the impact of climate change exposure on mental health outcomes^6^. The 8 studies related to interventions were primarily proposals and did not contain conclusive evidence. In another review on interventions for eco-anxiety, only 2 studies involved an empirical evaluation component, with the rest being conceptual or reflection papers^12^. The most comprehensive review to date identified 23 studies^13^, but did not differentiate between interventions implemented for a climate-related event and those for other settings (e.g., armed conflicts) theoretically relevant to the climate change context. The review was further limited to academic literature and interventions for preventing or treating known psychiatric disorders. As the World Health Organization (WHO) defines mental health as not the mere absence of a disorder but an overall state of wellbeing^14^, a broader conceptual framework could be helpful to recognize interventions that promote psychological strengths and emotional resilience in the face of climate stressors.

As increasing numbers of mental health interventions are being formally or informally developed and implemented in the context of climate change, there is a strong need to map out this space to facilitate knowledge exchange and identify best practices to scale-up support. Indeed, one of the global research priorities identified in a recent consensus building exercise is to “assess the appropriateness, feasibility, effectiveness, and scalability of mental health and psychosocial interventions (clinical and non-clinical) in the context of climate change”^15^. The current review aims to address this need by (1) focusing on existing interventions aimed at promoting mental health or mitigating the impacts on mental health; (2) considering broad mental health and wellbeing outcomes, which are not limited to predefined psychiatric diagnoses or newly described psychological states; and (3) incorporating a formal gray literature search process to identify interventions that have not been studied academically but are being implemented. The exploratory and flexible nature of a scoping review represents, therefore, an ideal methodology. To our knowledge, this is the first scoping review that aims to identify all such mental health and psychosocial interventions that have been implemented in the context of climate change.

## Methods

The scoping review was conducted in accordance with the JBI Manual for Evidence Synthesis^16^. The protocol was registered prospectively on March 9, 2022 and is available at: https://osf.io/dya94/.

### Inclusion criteria

The scoping review included records describing existing interventions that promote mental health or mitigate mental health impacts in the context of climate change. No restrictions were imposed on intervention target populations or geographical settings. All study designs were considered, given the a priori understanding that many interventions likely have not been formally evaluated through clinical trials. Further, given the interdisciplinary nature of the topic, records in the humanities and applied social sciences may not be easily categorizable into a study design conventional to medicine or public health.

### Exclusion criteria

The scoping review excluded records describing interventions for humanitarian crises not directly related to climate change (e.g., earthquakes, nuclear disasters, acts of terrorism), or natural hazards (e.g., recurrent floods) not explicitly connected with climate change by the authors. Other ineligible records included general resources (e.g., videos, webinars, podcasts, newsletters, therapist directories) that do not have an active interventional or participatory component; private, stand-alone retreats or courses; and books or book chapters. Proposals and expert opinions on psychotherapeutic approaches, while valuable to inform intervention development, are not organized programs that have been expanded beyond the individual therapist’s practice or systematically evaluated or implemented; they were therefore excluded.

### Academic literature search strategy

The academic literature search strategy combined three core constructs, “intervention”, “mental health” and “climate change”, the definitions of which are outlined in Table 1. The search terms were first developed for MEDLINE (Supplementary File 1) and modified for other databases as required. MEDLINE, PsycINFO and Web of Science were carried out from inception to May 2, 2022. Reference lists of any included studies and relevant reviews were hand searched to identify additional articles.

**Table 1:** Core conceptual constructs of the scoping review and their definitions.

Construct	Definition	Examples
Intervention	“Any type of local or outside support that aims to protect or promote psychosocial well-being and/or prevent or treat mental disorder”^74^	Psychological therapies, community-based programs, activism-based activities (e.g., Climate Cafés)
Mental health	“Mental health is a state of mental well-being that enables people to cope with the stresses of life, realize their abilities, learn well and work well, and contribute to their community”^75^	Psychological distress, symptoms of psychiatric disorders, suicide and suicidal behaviors, psychological responses to climate change (e.g., climate anxiety, ecological grief), positive mental health outcomes (e.g., resilience)
Climate change	“A change of climate which is attributed directly or indirectly to human activity that alters the composition of the global atmosphere and which is in addition to natural climate variability observed over comparable time periods”^76^	Global warming, extreme weather events, rising sea levels

### Study selection

All identified citations were collated in EndNote X9 with duplicates removed, then uploaded into Rayyan.ai, an online tool for conducting systematic and scoping reviews^17^. Titles and abstracts were screened by one reviewer (SX), with a subset (10%) screened by a second reviewer (SAK). Full texts of potentially relevant studies were acquired and screened by SX, a subset (10%) of which was also screened by SAK. Any discrepancies in reviewer decisions were resolved via discussions with a third reviewer (EL/AM).

### Gray literature search strategy

A comprehensive gray literature search was undertaken to capture interventions not published in peer-reviewed journals or evaluated in academic literature. The search strategy was informed by the methodology and best-practice guidelines by Godin et al.^18^ and Pollock et al.^18,19^, and involved four distinct steps: (1) targeted database search; (2) Google search; (3) targeted website search; and (4) key stakeholder consultation. The first three steps were carried out in May 2022 and the consultations took place between June-July 2022. Details of the gray literature search strategy can be found in Supplementary File 2.

### Data extraction

For academic literature, data were collected concerning the records (authors, publication year, study design) and nature of the interventions (delivery country/location; target population; climate stressor; mental health and wellbeing outcomes; evaluation outcomes; and any other available details, such as theoretical framework, intervention length, cost, facilitator characteristics, delivery methods, and involvement of stakeholders in co-designing the intervention). Best efforts were made to extract the same categories of data from gray literature. As the content from organizations may be trademarked, direct quotations from websites were at times extracted to best reflect the intervention description as intended. Data were presented in tabular form and analyzed narratively in accordance with their relevance to the review objectives.

### Data presentation

Results are presented by clustering interventions according to Bronfenbrenner’s ecological theory^20,21^. The theory describes one’s ecological environment at four levels: microsystem (individual and immediate home environment); mesosystem (peer groups and social networks); exosystem (institutions, the media, and local government), and the macrosystem (policies, laws, and overarching social structures). Bronfenbrenner’s theory has been widely applied in public mental health research given its embracing of the complex interplay between individual and contextual factors. We believe categorizing the interventions at the different levels would allow for a clear conceptual map of where work has been done and where more work is needed, and the presentation of most pertinent information to relevant stakeholders (e.g., policymakers).

## Results

### Overview of academic literature findings

**Fig. 1:**
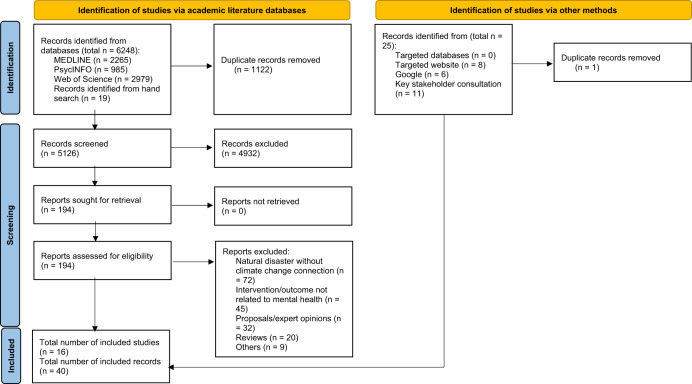
PRISMA flow diagram.

In total, 5126 unique records were identified (5107 from databases and 19 from backward citation hand search) (Fig. 1). Among the 194 records reviewed in full, 16 met inclusion criteria and were included in the review (Table 2). The 16 studies described 13 unique stand-alone interventions or packages of interventions across Asia, Europe, North America and the Caribbean, Oceania, and Sub-Saharan Africa. Among the studies, 7 (44%) were conducted in LMIC settings. All studies were published in or after 2009, with half (50%) having been published within the past 3 years (2019–2022).

**Table 2:** List of records identified through academic literature search (*n* = 16).

Author; year	Level of action	Location	Study design	Climate stressor	Target population	Intervention details	Co-design?	Mental health outcome (measure)	Evaluation results
Buchs et al. 2015	Mesosystem	United Kingdom	Cross-sectional	Climate change	N/A	Guided group sessions (typically 6 sessions with 6–8 individuals per group, moderated by 2 trained volunteer facilitators) with themes set out in the handbook; created by Rosemary Randall and Andy Brown	N/A	Emotional responses from participating in Carbon Conversations (semi-structured interviews)	Participants reported feeling less scared, less powerless, and more empowered (greatest perceived benefit among those with interest in climate change but has not engaged deeply in addressing carbon footprint)
Coppock et al.^38^	Macrosystem	Ethiopia (Liben and Moyale Districts)	Quasi-experimental	Multi-year droughts; increased soil erosion	Pastoral communities	(1) Step-wise capacity-building interventions (59 collective-action groups with total membership of 2300) ( 2) Livestock trading grant	Yes	Confidence in the future; quality of life; human health (study-specific survey)	Capacity-building package plus trading grant improved personal/household wellbeing attributes in both Districts in comparison to control group
Ede et al.^22^	Microsystem	Nigeria (Kogi state)	RCT	Floods	Flood victims with clinical depression	REBT (20 sessions; 50 minutes each) delivered in a group setting by therapists with PhD in career/mental health rehabilitation counseling	N/A	Depression (HDRS, Goldberg Depression Scale)	Intervention group had significantly decreased depression symptoms in comparison to waitlist control group
Gibson et al.^26^	Microsystem	Tuvalu (Nui Island and Funafuti Island)	Quasi-experimental	Cyclone Pam; “impending threat of climate change”	Adult islanders with mild-to-moderate symptoms of anxiety, depression, and/or PTSD symptoms resulting in functional impairment	Skills for Life Adjustment and Resilience (SOLAR) program in a group setting (up to 10 participants per group) over 5 consecutive days, delivered by trained non-specialist facilitators or ”coaches”	Yes	Psychological distress (HSCL-25), PTSD (PCL-5), impact of psychological distress on daily functioning (TIC); psychological impact from self-identified difficulty (PSYCHLOPS)	Participants had significantly decreased distress/post-traumatic stress symptoms and functional impairment after the intervention, with some effects retained at 6-month follow-up
Gros et al.^37^	Macrosystem	Bangladesh (Brahmaputra river basin)	Quasi-experimental	Monsoon floods	Poor households in vulnerable communities	Red Cross Red Crescent Project distributed flood-forecast-based unconditional cash transfer (USD 60 equivalent)	Yes	Psychological distress (3-question survey based on PSS)	Intervention group was less likely to experience psychological distress after the flood or feel anxious/depressed in the last seven days before the survey
Hart et al.^35^	Exo-system	Australia (New South Wales)	Case study	Drought, flood, fire, climate change	Rural farming communities affected by drought	Various; dedicated full-time drought mental health workers; farmer with lived experience/ RAMHP based on DMHAP with new components targeting aboriginal communities, older farmers, youth, women and substance use	Yes	N/A	N/A
Hechanova et al.^25^	Microsystem	Philippines (three communities in Samar province)	Pre-post	Super Typhoon Haiyan	Adult Haiyan survivors	Locally adapted “Katatagan” resilience intervention delivered in a group setting (5–7 participants per group) over 2 days as part of multi-day mission trips that provide medical/dental and social services	Yes	Coping self-efficacy based on learning objectives of each intervention module (study-specific survey based on module domains)	Participants improved in coping self-efficacy in all module domains managing unproductive thoughts and emotions and identifying personal strengths
Hechanova et al.^24^	Microsystem	Philippines (Tacloban City in Leyte province)	Quasi-experimental	Super Typhoon Haiyan	Adult Haiyan adult survivors	Locally adapted “Katatagan” resilience intervention delivered in a group setting (8 participants per group) by trained paraprofessionals	Yes	Anxiety (as measured by STAI-AD); adaptive coping (Brief Cope Survey); individual resilience (CD-RISC)	Intervention group had reduced anxiety scores and increased individual resilience 7–8 months post-intervention in comparison to control group; improvement in adaptive coping was less sustained
Heinz et al.^33^	Exo-system	United States (Sonoma County, California)	Cross-sectional	Wildfire	Community members affected by wildfire	Sonoma Wildfire Mental Health Collaborative: (1) Free trauma-informed yoga and meditation classes facilitated by trained yoga instructors ( 2) SPR training to counselors and paraprofessionals	Yes	“Feeling better” after each class (study-specific survey)	Most participants (84%) reported feeling better after class; repeat attendees reported feeling better for the rest of the week (32%), “lasting effects at reducing heightened response to ongoing stressors and episodic triggers”. Limited data to conclude SPR was associated with any mental health improvement
Heinz et al.^34^	Exo-system	United States (Sonoma County, California)	Multiple-baseline single-case experiment	Wildfire	Adolescents aged 13–17 years directly impacted by the wildfire with PTSD symptoms	Sonoma Wildfire Mental Health Collaborative: “Sonoma Rises” mental health app based on SPR and uses select audio tools from PTSD Coach	Yes	Daily ratings of anxiety/fear; PTSD symptoms (CPSS-5); internalizing/externalizing symptoms (BFS); psychosocial functioning (OSY); anxiety (GAD-7); depression (PHQ-9); wellbeing (WEMWBS)	No significant effects on clinical/functional outcomes detected; may be due to confounders/small sample size
James et al. 2019	Microsystem	Haiti (Port-au-Prince)	RCT	Various natural disasters (hurricanes, flooding, landslides)	Vulnerable community members	3-day mental health integrated disaster preparedness intervention in a group setting (up to 20 participants per group) delivered by trained Haitian lay mental health workers	N/A	PTSD (MPSS); anxiety (BAI); depression (ZLDSI); functional impairment (adapted local instrument)	Intervention group had decreased mental health symptoms and functional impairment from baseline; and exhibited a trend in increase in social cohesion
Koger et al.^11^	Microsystem	United States (New York City)	Narrative review	Climate change	People “tired of waiting for legislative action for climate change”	Environmental Health Clinic (structured problem-based coping)	N/A	Anxiety and concern about environmental issues	N/A
Nabhan et al.^30^	Mesosystem	United States (Arizona-Mexico border)	Narrative review	Climate change	Anglo and Hispanic youth aged 13–19 from low-income households	Borderlands Earth Care Youth Institute (hands-on nature restoration work); essays and reflections on land ethics and nature	N/A	Emotional strength (study-specific survey)	Internal program evaluation demonstrated positive effects of the program including improved emotional strength
Patrick & Capetola,^31^	Mesosystem	Australia (Victoria state)	Case series	Climate change and regional food insecurity	Hard-to-reach groups (individuals who are socially isolated, of low SES, public housing residents and people with chronic and mental health illnesses)	Community garden hub and many associated programs, including community kitchen, market, school gardening and agricultural courses, tree-planting workshops, and sensory garden for hospital patients and aged-care residents	Yes	General mental health wellness (unknown measure)	Internal program evaluation demonstrated improvements in mental health and social connectedness for participants
Richards et al.^32^	Mesosystem	Canada (Yukon Territories)	Commentary	Climate change affecting salmon numbers	Selkirk First Nation communities who rely on fish camps	On-the-land activities at fish camp for youth to connect with indigenous traditional knowledge facilitated by local community members including Selkirk Elders; participatory research documenting climate impact	Yes	Mental, emotional, and spiritual wellbeing	N/A
Tonna et al., 2009	Exo-system	Australia (New South Wales)	Case study	Drought	Rural farming communities affected by drought	Various; dedicated full-time drought mental health workers and staff trained in mental health triage and rural issues for support line; DMHAP with core components including community health forums and rural telephone support line	Yes	N/A	N/A

*BAI* Beck Anxiety Inventory, *BFS* Behavior and Feelings Survey, *CD-RISC* Connor-Davidson Resilience Scale, *CPSS-5* Child PTSD Symptom Scale for DSM-5, *DMHAP* Drought Mental Health Assistance Package, *GAD-7* General Anxiety Disorder-7, *HSCL-25* Hopkins Symptom Checklist-25, *HDRS* Hamilton Depression Rating Scale, *MHFA* mental health first aid, *MPSS* Modified PTSD Symptom Scale, *OSY* Ohio Scale for Youth Functioning Subscale, *PCL-5* PTSD Checklist for DSM-5, *PHQ-9* Patient Health Questionnaire-9, *PSYCHLOPS* Psychological Outcome Profiles, *PSS* Perceived Stress Scale, *PTSD* post-traumatic stress disorder, *RAMHP* Rural Adversity Mental Health Program, *REBT* rational emotive behavior therapy, *RCT* randomized controlled trial, *SES* socioeconomic status, *SPR* Skills for Psychological Recovery, *STAI-AD* State-Trait Anxiety Inventory for Adults, *TIC* Tuvalu Impairment Checklist, *WEMWBS* Warwick-Edinburgh Mental Well-Being Scale, *ZLDSI* Zanmi Lasante Depression Symptom Inventory.

Climate stressors included general climatic change, wildfires, droughts, cyclones, typhoons, and floods. Targeted mental health outcomes included psychological distress, psychiatric symptoms (depression, anxiety, PTSD), and broader psychological wellbeing measures such as emotional strength, emotional self-efficacy, confidence in the future, and general mental and spiritual wellness. Nine (56%) studies involved a type of design (e.g., RCT, quasi-experimental, pre-post) aimed to quantitatively evaluate the interventions, and 7 (44%) reported effectiveness results. Eleven (69%) studies mentioned a co-design process, during which local stakeholders were consulted for the needs assessment, intervention design, and/or cultural adaptation of the intervention.

### Description of interventions from academic literature

Five microsystem interventions were identified that primarily focus on individual-level emotions, behaviors, and psycho-emotional resilience.

In Nigeria, where increasingly frequent floods are being attributed to climate change, an evaluation of a rational emotive behavior therapy (REBT) program was conducted^22^. REBT is a short-term therapy related to cognitive behavioral therapy (CBT) and encourages participants to actively dispute irrational beliefs after experiencing an undesirable triggering event. REBT was delivered over 20 sessions to 49 flood victims with clinical depression by professional therapists. In comparison to the waitlist control group, the intervention group was found to have significantly decreased depression symptoms as measured by the Hamilton Depression Scale (*F*(1,97) = 208.935, *p* = 0.001, ηp^2^ = 0.69) and by the Goldberg’s Depression Scale (*F*(1,97) = 34.842, *p* = 0.001, ηp^2^ = 0.27) and at 3-month follow-up.

In Haiti, a disaster-prone country susceptible to climate change effects, an integrated community intervention was developed to promote mental health and improve practical disaster preparedness^23^. The intervention was manualized and consisted of activities that promote mental health literacy and coping skills (e.g., progressive muscle relaxation), and increase household-level preparedness (e.g., developing emergency action plan, mapping community risks and resources). The intervention was delivered by local lay workers over 3 days to 144 individuals who recently experienced a hurricane and associated flooding. In comparison to the control group, intervention participants experienced fewer depression (*B* = −0.35, *p* < 0.001), anxiety (*B* = 0.27, *p* < 0.001), and PTSD (*B* = −0.46, *p* < 0.001) symptoms, and increased mental health-focused help-giving intention (*B* = 2.62, *p* < 0.001).

Several interventions identified themselves as resilience-building programs. In the Philippines, a six-module intervention *Katatagan* was developed in the aftermaths of the Super Typhoon Haiyan and evaluated in two settings^24,25^. In Tacloban City where trained paraprofessionals delivered the intervention, the 48 intervention participants experienced lower anxiety scores (*F*(1,105) = 3.89, *p* = 0.05, ηp^2^ = 0.036), increased adaptive coping (*F*(2.79,192.6) = 5.87, *p* = 0.001, ηp^2^ = 0.078), and increased individual resilience (*F*(1,105) = 4.68, *p* = 0.03, ηp^2^ = 0.043)^24^ in comparison to the control group. In the Samar Province, the intervention was delivered as part of a mission trip by Health Futures Incorporated (HFI). The 163 intervention participants improved in all self-efficacy domains, as measured by a locally constructed scale based on each *Katatagan* module, in comparison to the participants’ baseline. Reported effect sizes ranged from Cohen’s *d* = 0.33 for Engaging in Positive Activities, Cohen’s *d* = 0.51 for Managing Thoughts and Emotions, to Cohen’s *d* = 0.83 for Seeking Solutions and Support^25^. Benefits were largest immediately post-intervention and decreased at six-month follow-up in both settings.

Skills for Life Adjustment and Resilience (SOLAR) is another resilience-building intervention that was piloted in Tuvalu, a small island developing state (SIDS) vulnerable to sea-level rise^26^. Forty-nine islanders impacted by Cyclone Pam and experiencing mental health symptoms participated in the lay-worker-delivered group intervention over 5 consecutive days. Module content included Skills for Healthy Living, Managing Strong Emotions, Getting Back into Life Following Disaster, Coming to Terms with Disaster, Managing Worry and Rumination, and Maintaining Healthy Relationships. In comparison to controls, participants experienced improvements in psychological distress (Glass’s *d* = 1.106), PTSD symptoms (Glass’s *d* = 1.575), and functional impairment (Glass’s *d* = 1.316). Benefits were retained at six-month follow-up but were reduced compared to immediate post-intervention.

One review study narratively described an Environmental Health Clinic based at New York University^27,28^. Individuals concerned about environmental issues received “prescriptions” to participate in environmental projects, with the aim to channel anxiety to specific climate action. This intervention was used as an example of structured problem-based coping and no evaluation was available.

Four mesosystem interventions were identified that involve focuses beyond individual-level changes, and additionally target peer group relationships and local community identity and cohesion in relation to mental health and wellbeing.

Carbon Conversations is a UK-based third-sector initiative which allows individuals to reflect on difficult emotions around climate change, and in turn be better able to engage with carbon footprint reduction^29^. Groups of six to eight participants meet with two facilitators over six sessions and discuss themes pertaining to Climate Change and Low Carbon Futures, Energy in the Home, Travel and Transport, Food and Water, and Consumption and Waste. In an online survey to 113 group participants, 50% agreed or strongly agreed that taking part helped them “face their worries about climate change”. Semi-structured interviews further revealed themes that the intervention allowed participants a unique space to express and share difficult emotions around climate change, feel more empowered and in control, and engage with others with similar experiences.

In various parts of the world vulnerable to extreme weather and regional food security, multiple interventions involving community-level participatory activities have been developed. These include: (1) land restoration work and associated reflection exercises for youth living at the Arizona-Mexico border in the United States; (2) community garden hubs with tree-planting activities for low-income residents and people with chronic and mental health conditions in Australia; and (3) traditional fish camp activities and participatory research for indigenous youth of the Selkirk First Nation in Canada^30–32^. All interventions were designed in the context of climate change to improve psychological and/or spiritual wellbeing, and to promote connectedness among participants through shared identities. Internal program evaluations of the land restoration and community garden interventions both anecdotally suggest improved mental health outcomes among participants, though the specific evaluative methods and outcome measures were not reported.

Two packages of exosystem interventions were identified that involved the implementation of multi-pronged mental health services and mobilization of the media and local institutions.

In Sonoma County, California, the Sonoma Wildfire Mental Health Collaborative was established following the historic 2017 wildfires^33,34^. The Collective launched a package of interventions that included a trauma-informed yoga and meditation program, a mental health app targeting adolescent survivors, and a Skills for Psychological Recovery (SPR) training program for counselors and paraprofessionals. The package was coupled with a media campaign to raise post-disaster mental health awareness, destigmatize help-seeking, and promote available resources. Given small sample sizes, the evaluative study was unable to conclude the effects of the app or SPR training; a preliminary survey suggested that participants experienced short-term beneficial effects from the yoga and meditation program.

For the rural farming populations who face climate-related adversity in New South Wales, Australia, a government-funded Drought Mental Health Assistance Package (DMHAP) was implemented^35,36^. DMHAP consisted of mental health promotion (e.g., resource booklet development, community mental health forums, mental health first aid trainings) and early intervention (e.g., rural telephone support line, service network planning workshops). Following renewed funding, the extended Rural Adversity Mental Health Program (RAMHP) increased the number of dedicated drought mental health workers and introduced specific activities for priority groups, including women, youth, older farmers, and Aboriginal communities. The authors reported that given funding limitations, no formal outcome evaluation was carried out.

Two macrosystem interventions were identified that involved macro-level interventional components, primarily through poverty reduction to improve mental health and wellbeing outcomes.

In Bangladesh, a Red Cross Red Crescent humanitarian project was delivered to promote financial security and associated psychological benefits among river basin communities amidst the 2017 floods^37^. A forecast-based unconditional cash transfer of BDT 5000 (USD 60 equivalent) was distributed to 1039 poor households prior to a flood peak. Relative to the unassisted households, intervention households were less likely to have always felt anxiety and depression (43% vs 29%, *p* = 0.015) and less likely to have always felt miserable or unhappy (61% vs 40%, *p* < 0.01) since the flood. While these findings were triangulated with qualitative interviews, the authors noted that the intervention benefits were not sustained after a second flood peak in the same year.

In Ethiopia, a capacity-building intervention was developed for pastoral communities who face increasingly severe droughts, land changes, and food crises. The intervention included components to inspire motivation, build collective-action groups, and improve literacy and numeracy^38^. The macro-economic components specifically involved the promotion of microenterprises and distribution of donor grants for livestock trading. The study reported the creation of 59 primarily women-led collective-action groups, 11 of which received donor grants. Following a major drought, intervention participants who received both capacity building and donor grants in the Liben District scored much higher on study-defined mental health and wellbeing attributes than their peers. The attributes included better ability to recover from crisis (OR = 91.7, *p* < 0.001), more confidence in the future (OR = 33.6, *p* < 0.001), and better human health (OR = 19.2, *p* < 0.001). The positive findings were replicated in the Moyale District with smaller effect sizes.

### Overview of gray literature findings

Targeted database search, Google search, and targeted website search identified 14 records describing 14 organizations offering relevant stand-alone and/or packages of interventions. One of the interventions (Carbon Conversations) was already identified through academic literature. A list containing these interventions, along with the inclusion and exclusion criteria, was circulated to 30 international content experts for consultation; through snowballing, we were introduced to and contacted 6 additional experts. Of all individuals contacted, 26 (72%) responded to the gray literature search request, and 8 (31%) of the respondents were based in a LMIC setting. The content experts reviewed and confirmed the list of interventions and identified 11 additional records for inclusion (total records = 25; minus duplicate = 24) (Table 3).

**Table 3:** List of records identified through gray literature search (*n* = 24).

Organization	Level of action	Location	Website	Climate stressor	Target population	Intervention details	Co-design?	Mental health outcome	Evaluation results
All We Can Save	Mesosystem	Global (based in the United States)	https://www.allwecansave.earth/circles	Climate change	Any	Self-organized groups for reading the book “All We Can Save” over 10 sessions (recommended 6–10 people per group); founded by Katherine Wilkinson and Ayana Johnson	N/A	Climate emotions	A survey for past participants is available to fill out on the organization website; results are not public
Circularity	Mesosystem	United States	https://circularitycommunity.com	Climate change	Organizations	Facilitation of in-person and virtual custom workshops that draw from climate psychology and nature therapy	N/A	Eco-anxiety symptoms	N/A
Climate Awakening	Mesosystem	Global (based in the United States)	https://climateawakening.org	Climate change	Any	Climate Emotions Conversations (group sharing and listening sessions; 4 participants per session) that occur 3 times per month guided by videos and conversation prompts; founded by Margaret Salamon	N/A	Climate emotions	N/A
Climate Café	Mesosystem	Global	https://www.climate.cafe (Numerous local websites available)	Climate change	Any	Informal community meetings for people to share climate-related feelings and inspire collective action	N/A	Climate emotions	N/A
Climate Cares guided journal	Microsystem	Global (based in the United Kingdom)	https://blogs.imperial.ac.uk/ighi/2022/03/01/what-can-we-learn-from-our-feelings-about-climate-change/	Climate change	Youth	Physical journal with 4-weeks of guided activity content to support a person’s “mental wellbeing and effectiveness in acting on environmental issues”; developed by Climate Cares	Yes	Climate emotions, general mental health and wellbeing	Positive qualitative comments from 40 youth who received the journal in a pilot study
Climate Psychology Alliance	Mesosystem	United Kingdom	https://www.climatepsychologyalliance.org/support/outreach	Climate change	Any	Therapeutic outreach program involving trainings and workshops on climate psychology and facilitated climate cafés	N/A	“Disturbing feelings, conflicts and dilemmas provoked by awareness of the climate crisis”	N/A
Conceivable Future	Mesosystem	United States	https://conceivablefuture.org	Climate change; US fossil fuel subsidies	Any (women-led)	House parties for individuals to connect, advocate against fossil fuel subsidies, and provide testimonies on the climate crisis, which is viewed as a reproductive justice crisis; led by Meghan Kallman and Josephine Ferorelli	N/A	Complex emotions towards reproduction and parenthood in the face of an unstable future and deteriorating environment	N/A
Deep Adaptation Forum	Mesosystem	Global (based in the United Kingdom)	https://www.deepadaptation.info	Climate change	“Persons over 18 who recognize the likelihood of societal collapse from climate change and environmental degradation”	In-person or virtual groups and recurrent events (nature and frequency dependent on facilitators); speaker and workshop offerings; founded by Jem Bendell	N/A	“Embody and enable loving responses to the predicament [societal collapse from climate emergency]”	N/A
Eco-Anxious Stories	Microsystem	Global (based in Canada)	https://ecoanxious.ca/	Climate change	Any	Online platform for climate and mental health storytelling; participatory “Sharing our Stories” worksheet, and services include eco-anxiety workshops, content creation and resource development; founded by Rachel Malena-Chan	N/A	“Complex range of emotions we feel about being alive right now”; “isolation and burnout as we navigate our role in making change”	N/A
Force of Nature	Mesosystem	United Kingdom	https://www.forceofnature.xyz/students	Climate change	Youth ages 16–25	Training programs for young people, youth speakers agency, student consulting network for businesses and non-profits, Anxiety-to-Agency workshops for students and educators; founded by Clover Hogan	Yes	Climate emotions, general mental health and wellbeing	N/A
Globe and Psyche	Mesosystem	Global (based in New Zealand)	https://www.globeandpsyche.com	Climate change	Professionals working in psychological, psychotherapeutic, and psycho-spiritual domains	Local conversation meetings to “explore what climate change means in their area, both its impacts and also opportunities for personal and collective healing”	N/A	“Personal, collective and climate stress”	N/A
Good Grief Network	Mesosystem	Global (based in the United States)	https://www.goodgriefnetwork.org	Climate change	Any	Group sessions (over 10 weeks) delivered by peers in-person or virtually based on the Alcoholics Anonymous Approach; co-founded by Laura Schmidt and Aimee Lewis Reau	N/A	“Painful feelings about the state of the world”	Internal evaluation suggested “participants report feeling less alone, more connected, empowered to take action in their lives”
Hold This Space	Microsystem	Global (created in the United Kingdom)	https://www.imperial.ac.uk/news/237137/new-website-helps-people-process-their/	Climate change	Any	An interactive website that guides individuals to “feel, imagine and connect” around climate change issues; developed by Common Vision in partnership with Climate Cares and Force of Nature	Yes	Climate emotions, general mental health and wellbeing	N/A
Ibanikom Climate Mental Health Literacy Project	Mesosystem	Cameroon	https://claretianuniversity.edu.ng/tag/ibanikom-health-literacy-evolution/	Flooding, landslides	Youth and older adults	A mental health literacy program built on Ibanikom ancestral and cultural identity and knowledge that involved meetings twice a week for 6 months; participants learned about the psycho-effects of climate change and co-developed local small-scale integrated health and agriculture projects that are ecologically sound	Yes	Eco-stress, eco-fatigue, eco-grief	One-year internal evaluation results indicative of community having increased awareness of climate disasters and mental preparedness of flood effects
One Earth Sangha	Mesosystem	Global (based in the United States)	https://oneearthsangha.org	Climate change	Any	Trainings, courses, and events aimed to build practices, community and action based on Buddhist tradition and Dharma teachings	N/A	“Wisdom, compassion, courage, creativity, flexibility, and a steady resolve to our inter-related ecological and social crises”	N/A
Project InsideOut	Mesosystem	Global (based in the United States)	https://projectinsideout.net	Climate change	Any	Online hub with interactive tools and resources to engage with and transform feelings, with the goal of becoming Guides to inspire changes in others	N/A	“Cycle between hope and despair, action and inaction, connection and disconnection”	N/A
The Climate Journal Project	Microsystem	Global (based in the United States)	https://www.theclimatejournalproject.com	Climate change	Climate enthusiasts, activists, and students	Live journal circles and weekly climate journal prompts to “cope with eco-anxiety, move past paralysis and transition into action”; founded by Yvonne Cuaresma	N/A	“Resilience against eco-anxiety”	N/A
The Eco-Anxiety in Africa Project (TEAP)	Mesosystem	Nigeria	https://www.teap.sustyvibes.org	Climate change	Young Africans	A project of Sustyvibes founded by Jennifer Uchendu; offers research service, community action events, and physical/virtual spaces for sharing climate emotions	N/A	“Eco-anxiety and environmental-related emotions in Africans”	N/A
The Resilience Project UK	Mesosystem	United Kingdom	https://www.theresilienceproject.org.uk/our-work	Climate change	Youth aged 16–23	Youth are trained through a residential program then lead 8-week Circles (typically 10 youth per Circle) to build knowledge and co-design programs to build resilience for other youth	Yes	Wellbeing, mental health, and resilience	N/A
The Resilient Activist	Mesosystem	Global (based in the United States)	https://www.theresilientactivist.org	Climate change	Any	Self-care, speaker’s bureau, online events, climate cafés, and nature-connected programming that support emotional wellbeing; founded by Sami Aaron		“Build resilience, optimism, and hope in response to the impact of the climate crisis”	N/A
The Rest of Activism	Mesosystem	Global (based in the United Kingdom)	https://www.climateemergence.co.uk/rest-of-activism-membership	Climate change	Any	A grant-subsidized program (by the Emergence Foundation) founded by Jo Musher-Sherwood that includes a weekly facilitated structured online space to support individuals’ “joy-filled activism”; monthly subscription fee required for membership	N/A	“Climate sorrow”, eco-anxiety, burnout	N/A
The Resource Innovation Group (TRIG)	Mesosystem	United States	http://www.theresourceinnovationgroup.org/transformational-resilience/	Climate change	Health professionals, emergency service providers, organizational and community leaders	Workshops, webinars, and conferences based on the Resilience Growth Model of Transformation	N/A	“Interlinked psychological and psychosocial-spiritual traumas and toxic stresses generated by climate disruption”	N/A
Transition Network	Mesosystem	Global (based in the United Kingdom and Europe)	https://transitionnetwork.org	Climate change	Any	Global network of community-led Transition groups that aim to build resilient communities and caring culture with an “Inner Transition” dimension (and “Heart & Soul” groups) that investigate the emotional/psychological aspects of climate action	N/A	“Feelings and sense of helplessness that often goes hand in hand with the reality of what is unfolding in our world today”	N/A
Work That Reconnects Network (WTR)	Mesosystem	Global (based in the United States)	https://workthatreconnects.org/	Climate change	Any	Forum, webinars, conversation cafés, workshops delivered by trained WTR facilitators; created based on work by Joanna Macy	N/A	“Despair and overwhelm”	N/A

All identified interventions are offered by an organization, and all but two were founded or based in a high-income country (HIC), primarily the US (46%) or the UK (33%). For the organizations that cited their founders, a large majority (11/12; 92%) of those founders are women.

The climate stressors addressed by interventions from the gray literature all involve general climatic changes and/or anticipated climate-related threats rather than specific climate-related disasters. Targeted mental health outcomes are independently defined by the organizations rather than by psychiatric diagnoses or standardized instruments, and many used emotion-based wordings such as “overwhelm”, “despair”, and “loneliness”. Five records (21%) described a co-design process involving local stakeholders.

### Description of interventions from gray literature

The 24 interventions or packages of interventions acted at the levels of microsystem and mesosystem. No exosystem or macrosystem interventions were identified through gray literature. Of note, none of the interventions reported formal evaluation methods; a minority (12.5%) reported selected positive internal evaluation results.

Four microsystem interventions were identified that take a self-guided approach and support individuals to improve their own mental health and wellbeing in the context of climate change.

In the UK, a group of organizations, namely Climate Cares based at Imperial College London, Force of Nature and Common Vision worked with young people and environmental scientists to create a virtual intervention Hold This Space^39^. The interactive website guides youth to explore their feelings towards climate change, imagine the world they would like to see based on the latest science, and reflect on how to act on environmental issues most concerning to them. In consultation with youth advisors, mental health practitioners, and climate change professionals, Climate Cares also co-designed a 4-week activity-based physical journal^40^. The goal is to reduce the mental health impacts that can be associated with climate-related distress, build coping strategies, envision a desired future, and increase capability to take desired action. The Climate Journal Project based in the US is another intervention involving a journaling approach^41^. The organization created digital and printed journals and worksheets that target eco-anxiety and environmental grief. Individuals have the additional option of participating in virtually guided “journal circles”. Other self-guided activities and worksheets were identified through Eco-Anxious Stories, a Canada-based online platform^42^. In the “Sharing Our Stories” worksheet, individuals are prompted to answer questions such as “Where is eco-anxiety showing up in my life?” and “What does a meaningful response to this crisis look like and feel like?”

The rest of 20 gray literature records all acted at the level of the mesosystem and involve a group-based or outreach approach that harness the power of group dynamics and community building.

Many of the interventions function on the premise of offering a safe space for individuals to gather and make sense of their positive or negative climate emotions. As a quintessential example, Climate Cafés are decentralized, drop-in meetings for discussing the climate crisis and building collective psycho-emotional resilience – often over tea or coffee. The model of Climate Cafés is now adopted by many organizations globally with both in-person and virtual meetings available^43–45^. The Good Grief Network developed an intervention based on the 12-Step approach of Alcoholics Anonymous^46^. Trained peer facilitators deliver the 10-week group program for individuals interested in recognizing and exploring their eco-distress and being supported to move towards meaningful action. The Network’s website reports that over 90% of program participants feel more empowered and less alone, though the exact survey methods or number of surveyed participants are not available publicly. Other examples of facilitated discussions include All We Can Save Circles (10 structured sessions)^47^, Climate Emotions Conversations by Climate Awakening (3 available sessions per month)^48^, and The Rest of Activism (2 available sessions per week)^49^. A few interventions name their specific target audience: Conceivable Future “house parties” are intended for individuals who wish to discuss reproductive decisions and parenthood while facing an uncertain future^50^, and Globe and Psyche conversation meetings are intended for individuals working in psychotherapeutic and psycho-spiritual domains to reflect on professional identities and healings for others^51^.

Five identified organizations offer packages of interventions (e.g., facilitated workshops, events, trainings, online communities) under the same theoretical or philosophical premise. The Work That Reconnects (WTR) is a network based on Joanna Macy’s work, also known as Deep Ecology Work and Active Hope. The work’s philosophical premise follows a spiral sequence of four stages, “gratitude”, “honoring our pain for the world”, “seeing with fresh eyes”, and “going forth”, and is designed to be delivered in an interactive group setting^52^. The Deep Adaptation Forum is based on Jem Bendell’s work^53^, which recognizes the “breakdown” from climate change and aims to support individuals to prepare for and co-create a loving response to what Bendell describes as the “inevitable near term societal collapse”. The Resilient Activist is based on the “Five Essentials” principle (Reconnect to Nature, Respect All Life, Regreen Our Planet, Revamp Our Spending, and Replenish Our Resources) to maintain a healthy mindset and ease the emotional burden from climate change^45^. The Transition Network offers a variety of interventions (e.g., “Heart and Soul” groups) based on the Inner Transition principle, which posits that shifts in emotional and psychological dimensions are needed to make outer systemic changes towards healthier communities^54^. One Earth Sangha is a hub for spiritual-psychological participatory groups and events in response to climate change based on Buddhist teachings^55^.

Seven interventions involve outreach or capacity-building approaches to improve the participants’ mental health and wellbeing and/or that of their wider community. Force of Nature runs training programs for youth affected by eco-anxiety. The trained youth then have the opportunity to run group “anxiety-to-agency” workshops, and become speakers or consultants for businesses and educators on matters related to climate change^56^. The Resilience Project UK also offers a program for young people, who then become leaders of an 8-week Circle to co-design resilience-building programs for other youth^57^. Project InsideOut is an online hub with interactive tools and resources, allowing individuals who experience climate emotions to undergo an inner transformation before becoming “Guides” for others and leading climate action^58^. Eco-Anxious Stories, Climate Psychology Alliance, Circularity, and The Resource Innovation Group (TRIG) all list eco-anxiety outreach services (e.g., speaker hub, trainings, workshops, resource development) for schools, organizations, and communities^42,44,59,60^.

Only two identified interventions at this level are based in a LMIC setting. In Nigeria, The Eco-Anxiety in Africa Program (TEAP) is managed by Sustyvibes - a non-profit climate activism organization. Reported TEAP activities include the creation of virtual and physical spaces to stimulate dialogs on climate change and mental health (e.g., “Sustyparties” that use poetry and open mic settings to facilitate the sharing climate emotions)^61^. In Cameroon, the Ibanikom Climate Mental Health Literacy Project facilitated meetings for flood-affected communities, allowing participants to learn about the effects of climate change on mental health and co-develop local, small-scale culturally relevant integrated health and agriculture projects^62^.

## Discussion

**Fig. 2:**
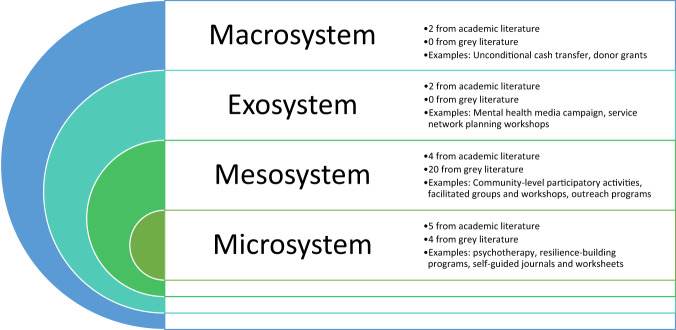
Identified interventions and their level of action based on Bronfenbrenner’s ecological theory as applied to public mental health research.

The impact of climate change on mental health and wellbeing is a pressing global challenge. More information is critically needed to plan for the design, implementation, and scaleup of effective interventions that address the dual and interconnected crises of ecological breakdown and mental health and psychosocial wellbeing burden. The present scoping review represents one of the first comprehensive efforts to fulfill this research priority and identified a total of 40 records which describe 37 unique interventions across academic and gray literature. The interventions acted at the levels of individuals, groups, local media and institutions, and larger social structures, and involved diverse mechanisms of action including psychotherapy, resilience-building programs, nature-based activities, community strengthening networks, and climate activism projects (Fig. 2). The mental health and wellbeing targets included depression, anxiety, PTSD, emotional strength and resilience, and various climate emotions, stemming from both direct exposure to climate-related extreme events and awareness of climate change.

The academic literature search identified interventions implemented primarily in geographical areas at risk of extreme weather events, and the target populations were often residents of the areas. Interestingly, all studies that reported a formal evaluation methodology and intervention effectiveness results (*n* = 7) were conducted in a LMIC setting. Among these studies, 5 incorporated a co-design process, and all demonstrated promising initial results in reducing negative psychiatric symptoms and/or promoting positive mental health and wellbeing outcomes. It is well recognized that the climate crisis deepens pre-existing global inequities^63^; it is therefore encouraging to see that current research efforts have been attentive to supporting the most disproportionately affected populations. However, several studies with a longitudinal evaluation component commented on reduced interventional effects at follow-up^24–26^. Furthermore, it is common for the LMIC-based interventions to be funded or run by international humanitarian organizations (e.g., Red Cross, HFI) rather than being integrated into the local system, and the potential for the sustainable implementation and scaleup of these interventions remain less certain.

In contrast to the academic literature findings, the gray literature search identified mostly interventions delivered by organizations founded or based in HICs. Few interventions commented on a theoretical framework for the mechanism of intervention. None were formally evaluated or have evaluative data publicly available, and therefore it is not possible to determine whether they are effective in addressing their targeted mental health and wellbeing outcomes or have any unintended impacts. It is further difficult to draw the line between non-profit and for-profit organizations, as various workshops and events depend on out-of-pocket payments from participants. This highlights additional sustainability and accessibility issues for individuals who are intersectionally disenfranchised by financial insecurity and mental health burden and may also limit their potential scalability to low-resource settings.

Overall, it appears that conceptual linkage for interventions at the intersection of climate change and mental health remains at a nascent stage, and most interventions are newly designed with scarce or anecdotal evidence. Most of the existing trials involve microsystem-level interventions (i.e., targeting individual emotions and behaviors) implemented in LMIC settings. Even then, the interventions are limited to a single study or country, and the findings have not been replicated. While there are many mesosystem-level interventions that leverage group dynamics, the overwhelming majority are based in HICs and have not been academically evaluated. There is a shortage of exo- and macrosystem interventions that mobilize local government and media or incorporate socioeconomic reforms and policy changes that may influence downstream mental health and wellbeing outcomes, though there is clear evidence for the potential co-benefits of climate policies for mental health and wellbeing^1^. There is, in general, also a lack of publicly available implementation information (e.g., training procedure of facilitators, implementation cost) for existing interventions, which would be key for scaleup.

### Strengths and limitations

A strength of this review lies in its comprehensive conceptualization of mental health. To reflect that mental health is “an intrinsic part of our individual and collective health and wellbeing”^14^, the review included search terms such as “wellbeing”, “resilience” and “post-traumatic growth”. This open approach allowed us to identify interventions beyond the realms of clinical psychology or psychiatry and tap into fields such as international development, urban planning, and environmental public health. The resulting diverse findings suggest that climate mental health interventions are likely to require complex, multidisciplinary input. Another strength of this review is its incorporation of a rigorous gray literature review process, which involved a large panel of international content experts. The process allowed for the capturing of emerging interventions and those not formally evaluated. Moreover, the review sought to, beyond identifying interventions, determine which types of interventions are better supported by evidence, and where there are clear gaps for the evaluation of existing interventions and/or the design of new ones.

A limitation of the review is that the search terms were only in English, which likely influenced the outcome that all included academic literature articles were English-language ones. Potential relevant articles published in other languages describing local interventions may be omitted. Further, most of the identified gray literature interventions are based in HICs. While we purposively consulted content experts from LMICs, it is possible that the search was biased towards HIC-based content given the immediate professional network of the authors and given that HIC-based organizations are likely better resourced to host and publicize their interventions on websites. We recognize that there are likely many other local, grassroot initiatives that have not gained international traction, yet also provide safe, accessible, and community-relevant spaces for discussions and actions around climate change and mental health. Many Indigenous communities have historically and continue to implement cultural practices that care for the wellbeing of people and the non-human world. Additionally, without individually contacting the individual organizations, we cannot comment on whether the website information is accurate and up-to-date, or the scale of the intervention (e.g., how frequently the microsystem-level worksheets are being downloaded, how many individuals have participated in the mesosystem group interventions). Therefore, this review cannot claim to be an exhaustive search of all existing and active mental health and psychosocial interventions, but rather a best effort at describing and mapping out the present interventional space.

### Recommendations for future research

Most of the existing evidence supports microsystem-level interventions in LMIC settings. For these interventions, studies using more robust study designs and involving more participants, as well as studies involving their adaptation to other geographical regions, would be helpful to better evaluate their larger-scale effectiveness and scalability. Implementation science would need to be applied to better assess the feasibility, acceptability, cost-effectiveness, and sustainability of the interventions. Ideally, the studies would also include a process evaluation component to better elicit why longitudinal effects may be reduced. This review further identified various mesosystem-level interventions in HIC settings, and there is potential for these interventional models to be studied using robust trial methodologies.

Regarding the development and piloting of new interventions, there is much room to explore exo- and macrosystem interventions in both LMICs and HICs. In our literature search, we identified three studies that may offer important insight. All were secondary analyses of the social impacts of climate interventions, which originally did not have a mental health focus (and hence did not meet our inclusion criteria and were not summarized narratively in our results). In one study based in Zimbabwe, it was found retrospectively that a biogas project contributed to community cohesion and empowerment^64^; in another study based in China, planned relocation and sheltering was found to be protective against depression, anxiety, and PTSD symptoms among flood victims^65^. In contrast, a study conducted in six LMICs (Brazil, Cameroon, Indonesia, Peru, Tanzania, and Vietnam) concluded that the Reducing Emissions from Deforestation and Degradation (REDD+) initiative may have *negatively* impacted women’s wellbeing^66^. Suggested reasons for the wellbeing decline included unrealized expectations for REDD+ initiatives and limited advanced consideration of addressing gender inequality in REDD+ policies. The studies demonstrated that while macrosystem interventions aiming to improve social welfare and their larger environment likely offer additional psychological benefits, they may also have unintended consequences if mental health and wellbeing, and its relationship to factors such as participatory approaches and gender equity, is not considered at conception. The REDD+ study further highlighted the importance of involving underserved groups, such as women, in the intervention design, implementation, and decision-making stages, thereby empowering them with leadership roles and incorporating their own definitions and experiences of wellbeing. This point applies generally, and it is vital for the appropriateness and ultimately success of both the content and implementation of interventions that they are co-designed with the people for whom they are being created. Understandings of mental health and wellbeing, and the experiences of climate change vary across geographies and cultures, and it is important to not perpetuate colonial practices by imposing Western definitions and understandings universally^67,68^.

It has also been increasingly recognized that being in and feeling connected with nature is beneficial for human health, including mental health^69–71^. However, most of the existing studies are limited to cross-sectional assessments that outline the association between time spent in the natural environment and health benefits. Nature-based programs or eco-therapies, such as animal-assisted interventions, therapeutic horticulture, forest bathing, and social prescriptions that bring participants into nature do offer trial evidence, but many were developed or evaluated among specific populations (e.g., cancer patients, children), and have not yet been directly associated with climate change^72–76^. It would be of interest to further explore climate-informed nature engagement (e.g. “reciprocal restoration” or social prescribing interventions)^30^ and their mental health and wellbeing outcomes.

Finally, disaster psychiatry offers much knowledge of interventions for individuals and communities surviving potentially traumatic events, and many lessons can be drawn regarding what works, where, and for whom. It is likely that climate change has contributed to the development of many natural hazards (e.g., Hurricane Katrina) which have been the object of study in disaster psychiatry^77^. However, as developed interventions typically have not factored in climate change - an unprecedented ongoing and growing crisis - they would likely need to be adapted to support individuals to cope with not only current but future stressors, and incorporate strategies such as proactive, forecast-based interventions, and disaster preparedness. The interventions would also likely benefit from collaborating across multiple bio-psychosocial fields to work preventatively and to address contextual factors.

### Implications for policy and practice

Multiple studies identified in this review did not successfully carry out an evaluative component despite having the intent, primarily due to limited funding and/or resources to recruit a larger sample size. This reflects a need to invest in and better support the evaluation of mental health and psychosocial interventions in the context of climate change, such that resources can be channeled into best evidence-based practices. When these practices are identified, a streamlined process is needed for their integration into the local health and social care systems, particularly in low-resource settings using existing infrastructures such as community groups. Consideration of participation cost is crucial, such that individuals from all socioeconomic backgrounds can be included and benefit.

For the work that is being done informally or in a community-led manner, including the gray literature interventions identified here and emerging ones we are aware of anecdotally, should be convened, collated, and showcased in an accessible manner. For example, an “online hub” of currently available interventions and case studies of best practices could promote shared learning and evidence-based investment, while minimizing the likelihood of duplicated efforts^78^.

## Conclusions

There is growing awareness of the detrimental effects of climate change on mental health and psychosocial wellbeing. In response to this evidence-base and lived experiences globally, interventions have been designed to promote mental health and wellbeing as well as to manage the detrimental impacts. This scoping review identified interventions acting at the microsystem, mesosystem, exosystem, and macrosystem levels. While most interventions have not been evaluated, existing studies, primarily on protecting mental health in the context of climate-related disasters in LMICs, show preliminary promising results. More evaluative studies using robust trial designs are needed, especially those involving implementation research. Future interventions are recommended to consider at conception the definition of wellbeing, the interests of underserved groups, co-design, equitable access, and sustainability.

## Supplementary information

Supplementary Information
