# Supplementary material for: Mental health and psychosocial interventions in the context of climate change: a scoping review
Source: Npj Ment Health Res. 2024 Mar 12;3:10. doi: 10.1038/s44184-024-00054-1 (PMC10956015; doi:10.1038/s44184-024-00054-1)
Supplement: Supplementary file 1 — Supplementary Information [file 44184_2024_54_MOESM1_ESM.pdf]

Supplementary Information

Supplementary File 1: MEDLINE search strategy

**Construct**	**Search terms**
A. Climate change	Exp Climate/ OR Exp Climate Change/ OR Exp Greenhouse Effect/ OR Environmental Pollution/ OR Exp Global Warming/ OR climate change OR global warming OR greenhouse effect OR rainfall OR heat OR heat?wave OR extreme?cold* OR drought* OR snowmelt* OR flood* OR cyclone* OR hurricane* OR typhoon* OR sea?level OR wildfire* OR forest?fire* OR coast* erosion OR coastal change*.tw
B. Mental health	Exp Mental Health/ OR Exp Psychological Stress/ OR Exp Psychological Distress/ OR Exp Depression/ OR Exp Anxiety/ OR Exp Grief/ OR mood OR anxiety OR anxious OR depress* OR PTSD OR post?traumatic stress disorder OR anguish OR grief OR emotional distress OR emotional stress OR mental health OR mental disorder* OR mental ill health OR mental illness* OR mentally ill OR psychiatric diagnosis OR psychiatric disease* OR psychiatric disorder* OR psychiatric illness* OR psychiatric symptom* OR psychologic* distress OR psychologic* stress OR psychologic* disorder* OR well?being OR eco?anxiety OR climate anxiety OR solastalgia OR climate grief OR ecological grief OR resilien* OR post?traumatic growth).tw.
C. Intervention	Exp Program Development/ OR Exp Program Evaluation/ OR Exp Psychosocial intervention/ OR Exp Health Policy/ OR Exp Social Support/ OR Exp Social Welfare/ OR Exp Psychotherapy/ OR Exp Counseling/ OR intervention* OR psychotherap* OR psychoeducation OR counsel* OR mental health program OR psychosocial program OR community program* OR mental health service OR psychosocial service OR community service OR mental health support OR psychosocial support OR community support OR MHPSS.tw
Search = A AND B AND C	

Supplementary File 2: Grey literature search strategy

For targeted database search, the deliberate choice of Web of Science as one of the databases was to capture any relevant thesis dissertations for inclusion. In addition, two other grey literature-specific databases were searched: Policy Commons (policycommons.net) to identify policy briefs and organizational reports, and ClinicalTrials.gov to identify ongoing and/or unpublished trials. The latter two databases were searched using the combination of search terms “intervention”, “mental health” and “climate”. The titles of the first 100 results from Policy Commons and of all titles/abstracts from ClinicalTrials.gov were screened against the inclusion/exclusion criteria, with potentially relevant records reviewed in full.

Google search involved using the same combination of search terms “intervention”, “mental health” and “climate” as per the targeted database search. The “incognito” mode was used to minimize the influence of search history and preference on result generation. The first 10 pages or 100 results were screened by their title and brief text description underneath. Potentially relevant websites were reviewed in full.

Targeted website search was conducted for the websites of six well-recognized climate non-governmental organizations. These websites were Climate and Mind (www.climateandmind.org), Eco-Anxious Stories (ecoanxious.ca), Talk Climate (talkclimate.org), Climate Psychiatry Alliance (www.climatepsychiatry.org), Climate Psychology Alliance (www.climatepsychologyalliance.org), and Good Grief Network (www.goodgriefnetwork.org). The websites were reviewed in full, with particular attention paid to tabs describing the organizations’ associated programs and list of resources.

Based on findings from the above three steps, a draft list of psychosocial interventions identified through grey literature was generated. This list was circulated to a group of key stakeholders who are content experts in climate change, mental health, and intervention design and delivery; and who work in academia, non-profit organizations, and intergovernmental organization settings. The group was consulted through email and asked to review the draft list, identify any additional interventions they were aware of, as well as to identify other potential content experts. Best efforts were made to maximize the diversity of knowledge of the key stakeholders and to actively involve those based in low- and middle-income countries (LMICs).
